# Clinical Observation of Phacoemulsification Combined with Intravitreal Injection of Conbercept in Cataract Patients with Diabetic Macular Edema

**DOI:** 10.1155/2021/8849730

**Published:** 2021-02-05

**Authors:** Jing Wang, Yuqi Liu, Yiping Hu, Lu Lu, Kaili Tang, Jinsong Zhang

**Affiliations:** ^1^Shenyang Aier Exellence Eye Hospital, Shenyang, China; ^2^The Fourth Affiliated Hospital of China Medical University, Shenyang, China

## Abstract

**Aim:**

To observe the clinical efficacy and safety of phacoemulsification surgery combined with intravitreal injection of conbercept in cataract patients with DME.

**Methods:**

This is a prospective clinical cohort study. Thirty-five cataract patients (49 eyes) with DME were divided into two groups. The observation group (23 eyes) underwent a cataract phacoemulsification surgery combined with intravitreal injection of conbercept 0.5 mg; the control group (26 eyes) underwent a cataract phacoemulsification surgery only. The visual acuity, central macular thickness (CMT), IOP, and anterior chamber flare were examined before surgery and 1 week and one month after surgery.

**Results:**

The UCVA and BCVA in Log MAR in the observation group were lower than those in the control group at 1 week (*p*=0.032; *p*=0.041) and 1 month (*p*=0.035; *p*=0.039), respectively, after the surgery. The CMT of the observation group changed from 492.7 ± 32.2 *μ*m before surgery to 341.6 ± 59.9 *μ*m one week after surgery and 374.8 ± 48.3 *μ*m one month after surgery. The CMT of control group increased after surgery. There was no significant difference in IOP and flare between the two groups at all following times.

**Conclusion:**

In patients with DME, undergoing a cataract surgery combined with intravitreal injection of conbercept is safe and effective for visual improvement and CMT declination with relatively fewer IOP and flare fluctuation.

## 1. Introduction

With the increasing incidence of diabetes mellitus (DM) every year, China has now become the country with the highest number of diabetic patients in the world. In diabetic patients, due to systemic metabolic abnormalities and changes in blood glucose, cataracts will appear earlier than in patients without the disease. A diabetic cataract is also the main factor affecting vision in diabetic patients. Moreover, a cataract in diabetic patients will affect the diagnosis, treatment, and follow-up of fundus disease. Thus, diabetic patients need earlier cataract treatment and surgery than healthy patients. Considering that diabetes is a high-risk factor for macular cystoid edema after cataract surgery, the ocular trauma and inflammatory reaction of cataract surgery will aggravate the existing diabetic retinopathy (DR) and diabetic macular edema (DME) in diabetic patients. Therefore, the choice of treatment method and timing for cataract patients with DME is crucial for the long-term vision benefit of patients. At present, phacoemulsification surgery has been recognized as a conventional cataract treatment method. An intravitreal injection of an antivascular endothelial growth factor (anti-VEGF) drug can control retinopathy in patients, and it is the preferred treatment for DME. As a new recombinant fusion protein, conbercept (KH902) is a good choice for anti-VEGF drugs. Conbercept is a 143 kDa recombinant anti-VEGF fusion protein engineered from a full human cDNA sequence in Chinese hamster ovary cells [1]. Conbercept is composed of extracellular region two of VEGF receptor 1 (FLT 1) and extracellular regions three and four of FLT 2, which are fused with human immunoglobulin (IgG) Fc fragment. Conbercept is a multitarget, high affinity, 100% humanized recombinant fusion protein with a strong persistence. Conbercept has a very high affinity for VEGF, up to 0.1–0.3 pM, which can effectively capture VEGF and prevent VEGF-mediated activation of the angiogenesis pathway. At the same time, it can penetrate the retina completely and prevent the production of all subtypes of VEGF-A, VEGF-B, and placenta growth factor. This study intends to observe the clinical efficacy and safety of phacoemulsification surgery combined with intravitreal injection of conbercept in cataract patients with DME.

## 2. Materials and Methods

### 2.1. Subjects

This is a prospective clinical cohort study. The study protocol was approved by the medical ethics committee of the Fourth Affiliated Hospital of China Medical University (2015–028), in accordance with the Declaration of Helsinki. Between 2016 and 2018, cataract patients with DME who were planned to undergo phacoemulsification surgery in the cataract clinic of our hospital were selected. They were fully informed of the condition and treatment plan before surgery and gave written informed consent to be included. A total of 35 patients (49 eyes) were included, and they were divided into two groups according to the treatment plan selected by the patients. The observation group (23 eyes) underwent cataract phacoemulsification surgery combined with intravitreal injection of conbercept 0.5 mg; the control group (26 eyes) underwent only cataract phacoemulsification surgery. A piece of aspherical foldable intraocular lens (Tecnis ZCB00, Johnson and Johnson Vision) was implanted into the eyes of both groups. All patients were followed up with relevant eye examinations following the time specified in the trial to monitor the changes in the condition.

### 2.2. Inclusion and Exclusion Criteria

Inclusion criteria are as follows. ① All cases that were diagnosed by standard visual acuity chart, fundoscopy, fundus fluorescein angiography (FFA), and optical coherence tomography were included. Cataract patients with DME, cataract patients with lens nuclear of grade III (LOCS-III), and Spectralis OCT detection central macular thickness (CMT) higher than 300 *μ*m were included. FFA had to show that the stage of nonproliferative diabetic retinopathy (NPDR) was moderate or mild and combined with nonischemic macular edema. ② If both eyes met the inclusion criteria, the researchers would determine the target eye from the medical point of view. ③ Patients who had a history of type II diabetes, yet a stable blood pressure and blood glucose control during treatment and follow-up were included; the glycated hemoglobin control standard had to be less than 6.5%, and the premeal blood glucose was less than 8 mol/L; preoperative blood pressure should not have been higher than 160/90 mmHg. Exclusion criteria were as follows: ① patients who were not able to undergo FFA examination; ② those who had suffered from glaucoma, optic neuroretinopathy, uveitis, other macular diseases, and other eye diseases affecting visual function or macular morphology; ③ those who could not obtain the ideal OCT macular area scan due to refractive interstitial turbidity; ④ patients who had taken glucocorticoid therapy and other anti-VEGF drugs in the past three months; ⑤ patients with anterior segment disease; ⑥ patients with severe heart, brain, liver, kidney and hematopoietic system diseases, and allergies history; ⑦ patients with vitreous surgery and aphakia; ⑧ patients with a diopter higher than 6D; ⑨ corneal endothelial cells less than 1500/mm^2^.

## 3. Surgical Methods

### 3.1. Cataract Phacoemulsification Combined with IOL Implantation

Standardized phacoemulsification combined with intraocular lens implantation was performed in all patients by the same skilled doctor. Preoperative preparation was as follows: all patients were given levofloxacin eye drops (Santen, Japan) four times a day and bromfenac sodium (Senju, Japan) eye drops twice a day three days before operation. Thirty minutes before the surgery, tropicamide eye drops (Santen, Japan) was given three times to cause full mydriasis, and promethazine hydrochloride eye drops (Novartis, USA) were used to anesthetize the surface. A 2.2 mm tunnel incision was made over the temporal surface of the clear corneal; the auxiliary corneal puncture was made in the two-point direction, and the cohesive and dispersive viscoelastic composites DUOVISC (Alcon, USA) were then injected. Continuous circular and central capsulorhexis were performed, the diameter of which was about 5.5–6.0 mm. After water separation, the nucleus was removed by the liquid flow of the Centurion Phacoemulsification system (Alcon, USA) with Divide and Conquer method. After phacoemulsification of the nucleus and aspiration of the cortex, a piece of aspheric foldable intraocular lens (Tecnis ZCB00, Johnson and Johnson Vision), made of hydrophobic acrylate material, was implanted. The residual viscoelastic agent was removed, and a watertight incision was made.

### 3.2. Intravitreal Injection of Conbercept

Patients in the observation group were treated with intravitreal injection of conbercept immediately after cataract phacoemulsification and watertight incision. The needles were injected into the flat part of the ciliary body (3.5 mm behind the corneal limbus). After the needles were seen through the pupil, 0.05 mL conbercept (2.5 mg; produced by Chengdu Kanghong Pharmaceutical Group Co., Ltd., National Pharmaceutical Standard S20130012) was injected into the vitreous, and the injection point was pressed with a sterile cotton swab to avoid drug outflow. After the surgery, the conjunctival sac was coated with tobramycin dexamethasone eye cream and covered with sterile gauze. Postoperative medication was as follows: all patients began to routinely use tobramycin dexamethasone eye drops (Novartis, USA) four times a day, gradually decreasing within two weeks after operation. Within one week after operation, levofloxacin eye drops (Santen, Japan), four times a day, and compound tropicamide eye drops (Senju, Japan) for mydriasis, twice a day, were used. Bromfenac sodium (Senju, Japan) eye drops were given twice a day for one month after the operation.

### 3.3. Patient Examinations

The patients were assessed before surgery and then followed up at one week and one month after surgery. The vision, intraocular pressure (IOP), anterior segment, and fundus were examined (ChiCTR-IIR-15007619). All examinations were completed by the same ophthalmologist. (1) In terms of visual acuity, uncorrected visual acuity (UCVA) and best-corrected visual acuity (BCVA) were performed and recorded in the form of Log MAR. (2) In terms of CMT, frequency-domain OCT (Spectralis OCT, Heidelberg) was used for CMT detection when the pupil was fully dilated by compound tropicamide eye drops. 3D stereoscopic scanning of the macular area was performed, and the retinal thickness map was selected to record the thickness of the central macula. (3) In terms of IOP, a noncontact IOP measuring instrument (NT-530P, NIDEK) was used for IOP measurement. (4) In terms of anterior chamber flare, a Laser Flare Cell Meter (FM600, Kowa) was used to measure the aqueous humor protein concentration (Flare) in the state of natural pupils at a fixed time of 8:00–9:00 AM.

### 3.4. Statistical Analyses

IBM SPSS Statistics for Windows, Version 22.0, was used for analysis. The numerical data were expressed as *χ* ± *s*. For baseline data, a T-test and chi-squared (*χ*) ^*2*^ tests were used to analyze the differences between groups. The comparison of visual acuity, CMT, IOP, and flare between the two groups at different follow-up time points was performed using the T-test. The differences were considered statistically significant when the *p*-value was less than 0.05.

## 4. Results

### 4.1. Demographics

In this study, 35 patients (49 eyes) were included. There were 16 patients (23 eyes) in the observation group, including 7 males and 9 females, aged 49 to 77 years, with an average age of 58.4 ± 10.7 years. There were 19 patients (26 eyes) in the control group, including 9 males and 10 females, aged 52 to 81 years, with an average age of 60.2 ± 12.4 years. There was no statistically significant difference between the two groups regarding general data such as age, gender, DM course, preoperative BCVA (Log MAR), CMT, and IOP, as shown in Table 1.

### 4.2. Visual Outcome

**Figure 1:**
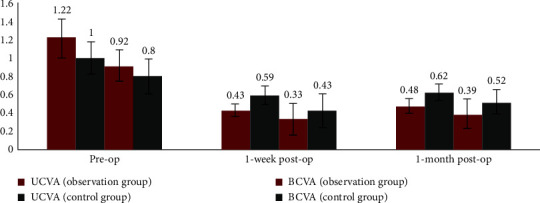
The trend of UCVA and BCVA at different time points in the observation group and control group.

Both groups of patients showed significant visual acuity improvement after surgery, as shown in Figure 1 and Table 2. The trend of visual acuity showed a significant decrease in Log MAR visual acuity after surgery. The UCVA in Log MAR in the observation group was lower than that in the control group, and there was a statistical difference between the two groups at one week (*t* = 2.364, *p*=0.032) and one month (*t* = 2.315, *p*=0.035) after surgery. Similarly, the BCVA in Log MAR in the observation group was lower than that in the control group, and the statistical difference was found between the two groups at one week (*t* = 2.229, *p*=0.041) and one month (*t* = 2.247, *p*=0.039) after surgery.

### 4.3. CMT

**Figure 2:**
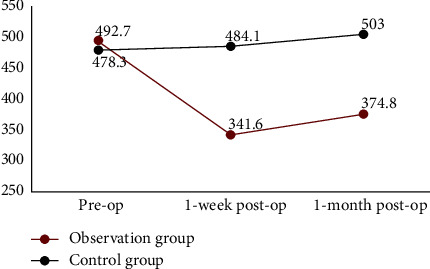
The trend of CMT at different time points between the observation group and control group.

**Figure 3:**
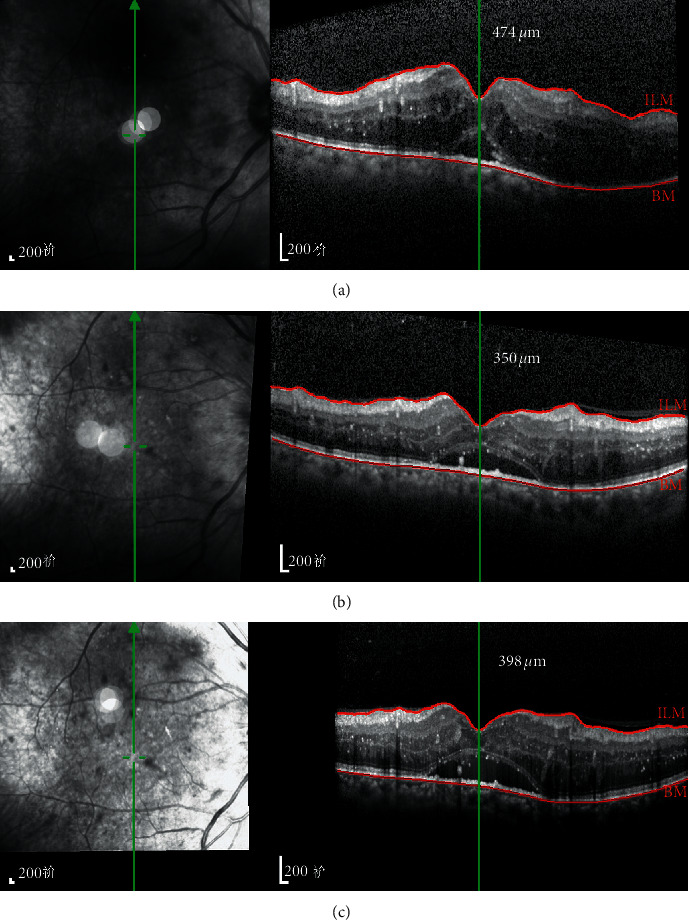
OCT tracking images of a 62-year-old male patient in the observation group before surgery and then at one week and one month after surgery. (a) Pre-op CMT = 474 *μ*m; (b) one week post-op CMT = 350 *μ*m; (c) one month post-op CMT = 398 *μ*m.

The CMT of the observation group changed from 492.7 ± 32.2 *μ*m before surgery to 341.6 ± 59.9 *μ*m one week after surgery and 374.8 ± 48.3 *μ*m one month after surgery. The mean CMT decreased by 151.1 *μ*m and 117.9 *μ*m, respectively, meaning that it was significantly lower than that before surgery, and the changing trend is shown in Figure 2. In the control group, the CMT was 478.3 ± 54.1 *μ*m before surgery; however, it increased at both one week and one month after surgery. The mean CMT increased by 5.8 *μ*m and 24.7 *μ*m, respectively. There were significant differences in CMT between the two groups at one week (*t* = 2.395, *p*=0.027) and one month (*t* = 2.365, *p*=0.032), which is shown in Table 3. The typical picture is shown in Figure 3.

### 4.4. IOP and Flare

Table 4 shows the IOP and flare at different time points between the observation group and the control group. After comparison, no statistically significant difference in IOP between the two groups was found before surgery or one week or one month after surgery (*p* > 0.05), which indicates the steady state of intraocular pressure during the perioperative period. After surgery, the average value of flare was higher, especially at one week; however, there was no significant difference between the two groups at different time points (*p* > 0.05).

## 5. Discussion

DME is one of the main causes of blindness due to diabetes; it can occur at any stage of the disease. A total of 10%–25% of diabetic patients have macular edema. Among diabetic patients who have severe retinopathy, the proportion of DME is higher. It is currently believed that DME is a complex pathological process involving multiple factors [2]. The main mechanisms are as follows: (1) the destruction of the inner blood–retinal barrier (BRB) caused by glial cells, pericytes, endothelial cells, and leukocytes; (2) the destruction of the outer BRB caused by damage to tight junction proteins; (3) the involvement of VEGF-A, protein kinase C, histamine, angiotensin II, matrix metalloproteinases, pigment epithelial factor, platelet-derived growth factor, basic fibroblast growth factor, and other vasodilators; (4) changes in the vitreoretinal interface caused by posterior vitreous detachment, posterior vitreous cortex liquefaction, premacular residual vitreous presence, increasing tension, pulling of the proliferative membrane, and increase in the internal limiting membrane and collagen crosslinking. These factors eventually lead to local retinal ischemia and increased vascular permeability in the macular region. As this pathogenic process progresses, it causes abnormal thickening and edema in the macular region.

Nowadays, it has been confirmed that VEGF plays an important role in the pathogenesis of DME. The severity of DME was positively correlated with the expression level of VEGF in patients' vitreous. The upregulation of VEGF expression can promote the expression of a variety of inflammatory factors, increase the permeability of retinal capillary, destroy the internal and external barriers of the retina, and lead to retinal edema and the occurrence and development of DME [3]. The intravitreal injection of anti-VEGF drugs has become the first-line treatment of DME due to their great efficacy in improving visual acuity and mitigating macular edema [4]. Other treatment methods include macular grid laser photocoagulation, micropulse laser therapy, and vitreous retinal surgery. As a new multitarget and high affinity recombinant fusion protein, conbercept has achieved good results in the treatment of macular edema caused by diabetic retinopathy, choroidal neovascularization of pathologic myopia (pmCNV), retinal vein occlusion (RVO), and wet age-related macular degeneration (wAMD) diseases [5–7]. Some studies, especially, have shown the clinical effect and safety of conbercept in the treatment of DME [4]. Zhou et al. found that conbercept was effective for visual and anatomic improvements in DME eyes with relatively fewer intravitreal injections and longer treatment intervals in clinical practice [8]. However, for the combined intravitreal injection in cataract patients with DME, there are currently no internationally and domestically recognized unified treatment guidelines, which is also an area of extreme interest in the interdisciplinary study of cataract and fundus diseases.

For patients with DME, cataract surgery should be considered when the clouded lens leads to a decrease of vision that delays fundus examination, treatment, and follow-up; these patients have earlier surgery needs than patients with simple age-related cataracts. However, it has to be considered that the mechanical and physical damage caused by surgical operations, residual lens after cataract surgery, foreign body reaction after IOL implantation, and the epithelial cell proliferation reaction will lead to increased production of prostaglandin in the eye. This will damage the blood–aqueous barrier and blood–retina barrier and finally lead to an inflammatory reaction after cataract surgery as it can manifest as iritis, fibrinoid exudation membrane, and cystoid macular edema (CME). Moreover, the inflammatory reaction may also aggravate DME. A study of 4850 diabetic patients by UK DR EMR [9] showed that the risk of treatment-requiring diabetic macular edema after cataract surgery was significantly higher than that before cataract surgery (2.9% vs. 5.3%), which had the greatest impact on severe NPDR. Yulin et al. also found that the central retinal thickness (CRT) of diabetic patients with NPDR and PDR increased significantly one month after cataract surgery, which was influenced by insulin treatment [10]. Diabetes mellitus and hypertension are some of the high-risk factors for increased CMT after cataract surgery. Therefore, for the patients who need cataract surgery with DME, it is necessary to consider the effect of possible further damage on the retinal barrier that can occur after surgery; the timing and surgical plan should be considered to reduce the risk of postoperative DME and DR. For cataract patients with DME, local application of glucocorticoids and nonsteroidal anti-inflammatory drugs during the perioperative period is essential for blocking the production of prostaglandins and leukotrienes and controlling the noninfectious inflammatory reaction after surgery. During the perioperative period, close attention should be given to blood glucose levels, incision healing, corneal epithelial integrity, and macular OCT morphological changes and performing FFA when necessary.

In this study, the observation group chose to perform cataract phacoemulsification surgery combined with intravitreal injection of conbercept. The visual acuity improved significantly at one week and one month after surgery. The difference was statistically significant compared with the control group. The postoperative CMT in the observation group decreased significantly, while that in the control group increased slightly. These results were close to those of Yumusak et al. [11]. Denniston et al. found that the concentration of VEGF in the aqueous humor of diabetic patients increased from 68 pg/ml at baseline to 723 pg/ml one day after surgery, yielding a tenfold increase [9]. However, it gradually recovered to 179 pg/ml one month after surgery, yet it was still around 2.5 times greater than that of the baseline value taken before surgery. Chae et al. showed that the combination of anti-VEGF intravitreal injection during cataract surgery in NPDR patients without DME could increase BCVA more, and CRT increased less [12]. As a new multitarget, high-affinity fusion protein anti-VEGF drug, conbercept can effectively reduce VEGF and prevent VEGF-mediated activation of angiogenesis pathways. Jampol et al. showed that the number of doses in the IVC group was significantly lower than that in the IVR group, while the frequency of administration of conbercept was less than that in the ranibizumab group, and the effect was better in patients with visual acuity less than 20/50 [13]. Therefore, the intravitreal injection of conbercept during cataract surgery was significant enough to control the increase of VEGF concentration, decrease the CMT, and control DME.

In this study, high-speed frequency-domain OCT was used for 3D scanning of CMT and CMT numerical analysis. HRT can automatically compensate for refractive errors. In many examinations, the images are arranged by software with vascular demarcation to eliminate slight eye movement and ensure the accuracy and objectivity of measurement results. However, the CMT of the control group was increased at one week and one month after surgery compared with that before the surgery, and the average increase of CMT at one month after surgery reached 24.7 *μ*m, which was higher than that of Cagini et al. for patients with simple age-related cataracts. The difference may be related to the destruction of the BRB in cataract surgery, increased intraocular inflammatory mediators, and increased risk of CMT after cataract surgery in diabetic patients [14]. DME was classified according to the morphologic pattern based on optical coherence tomography: diffuse retinal thickening (DRT), cystoid macular edema (CME), or serous retinal detachment (SRD). Due to the small sample size, this study did not divide patients into different groups with OCT patterns. Li et al. found that conbercept had a good effect on DME with different OCT types, especially the DRT group [15]. We need to do more further research.

This study also observed the main safety indicators of IOP and flare. In this study, a more objective assessment NT-530P-corrected IOP meter was used, which can eliminate the influence of corneal thickness on IOP. Quantitative measurement of the aqueous humor protein concentration can more objectively evaluate anterior segment inflammation after surgery. In this study, there was no significant change in IOP and flare, which reflects the safety of the combined surgery. For patients, the way of combined drug injection in cataract surgery can avoid the preparation process such as disinfection during the second surgery, reduce the stimulation and infection risk of repeated surgery, and reduce the surgery-related costs. Hence, patients have a better tolerance and higher acceptance, especially those with chronic systemic complications of diabetes. In this study, the aqueous humor protein concentration in the two groups after cataract surgery was higher than that before surgery, especially one week after surgery. However, no significant difference was found between the two groups in the flare, indicating that the risk of increased aqueous humor protein concentration was not increased by the combination of intravitreal injection during cataract surgery. The increased aqueous humor protein concentration at one week after surgery was consistent with the results reported by Chang. on the aqueous humor protein concentration in diabetic patients undergoing cataract surgery [16]. The rise of flare one week after the surgery directly reflected the degree of damage to the function of the blood–aqueous barrier as a result of the surgery. With the extension of time and the application of local anti-inflammatory drugs, flare recovered to the preoperative level at one month after surgery, reflecting the gradual repair process of the blood–aqueous barrier under the action of drugs.

In summary, it is safe for patients with DME to undergo cataract surgery combined with intravitreal injection of conbercept. Together, it can effectively reduce the CMT, improve UCVA and BCVA, reduce the number of surgeries, and improve the compliance of patients. However, due to the small sample size in this study, future investigations are warranted to explore the follow-up treatment plan of DME and explore whether to conduct continuous intravitreal administration and time of administration and whether to combine laser therapy and other treatment methods.

## 6. Conclusions

In patients with DME, combining catract surgery with intravitreal injection of conbercept is safe and effective for visual improvement and CMT declination with relatively fewer IOP and flare fluctuation.

## Figures and Tables

**Table 1:** Demographic profile and baseline characteristics of the patients.

Group	Age (years)	Gender (M/F)	DM course (years)	BCVA (Log MAR)	CMT (*μ*m)	IOP (mmHg）	Flare (pc/ms)
Observation group	58.4 ± 10.7	7/9	5.93 ± 1.66	0.92 ± 0.17	492.7 ± 32.2	14.1 ± 1.7	7.1 ± 2.1
Control group	60.2 ± 12.4	9/10	6.24 ± 1.97	0.80 ± 0.19	478.3 ± 54.1	13.8 ± 1.5	6.5 ± 1.9
*t*	0.849	0.694^∆^	0.327	−0.529	−0.458	−0.672	−0.764
*p*	0.402	0.539	0.745	0.671	0.683	0.573	0.417

^∆^ *χ* ^*2*^ test.

**Table 2:** Comparison of UCVA and BCVA at different time points between the observation group and control group.

	UCVA			BCVA		
	Pre-op	One week post-op	One month post-op	Pre-op	One week post-op	One month post-op
Observation group	1.22 ± 0.21	0.43 ± 0.07	0.48 ± 0.08	0.92 ± 0.17	0.33 ± 0.17	0.39 ± 0.16
Control group	1.00 ± 0.18	0.59 ± 0.10	0.62 ± 0.09	0.80 ± 0.19	0.43 ± 0.19	0.52 ± 0.13
*t*	−0.793	2.364	2.315	−0.529	2.229	2.247
*p*	0.427	0.032^*∗*^	0.035^*∗*^	0.671	0.041^*∗*^	0.039^*∗*^

**Table 3:** Comparison of CMT at different time points between the observation group and control group.

	CMT (*μ*m)		
	Pre-op	One week post-op	One month post-op
Observation group	492.7 ± 32.2	341.6 ± 59.9	374.8 ± 48.3
Control group	478.3 ± 54.1	484.1 ± 45.4	503.0 ± 41.7
*T*	−0.458	2.395	2.365
*p*	0.683	0.027^*∗*^	0.032^*∗*^

**Table 4:** Comparison of IOP and flare at different time points between the observation group and the control group.

	IOP（mmHg）			Flare（pc/ms）		
	Pre-op	One week post-op	One month post-op	Pre-op	One week post-op	One month post-op
Observation group	14.1 ± 1.7	14.4 ± 1.4	14.8 ± 1.5	7.1 ± 2.1	15.5 ± 1.4	8.9 ± 1.9
Control group	13.8 ± 1.5	13.9 ± 1.5	14.1 ± 2.8	6.5 ± 1.9	13.3 ± 1.2	8.1 ± 2.1
T	−0.672	−0.751	−0.716	−0.764	−0.944	−0.661
*p*	0.573	0.429	0.443	0.417	0.343	0.587

## Data Availability

The data used to support the findings of this study are available from the corresponding author upon request.
